# Supplementary material for: A multivariate analysis to propose linear models for the stature estimation in the Sabahan young adult population
Source: PLoS One. 2022 Aug 30;17(8):e0273840. doi: 10.1371/journal.pone.0273840 (PMC9426935; doi:10.1371/journal.pone.0273840)
Supplement: Attachment — Submitted filename: response to reviewers latest.docx [file pone.0273840.s003.docx]

Dear editor and reviewers,

Please accept our sincere gratitude for your volunteerism to review our manuscript to polish it to be fit for the renowned journal. We do appreciate your valuable time and critical thinking for the purpose to enrich the manuscript. Each of your comments are very crucial and we have tried our best either to incorporate into the manuscript or to answer to your queries.

The following are the responses prepared by the authors and have been inserted issue by issue:

Review Comments to the Author

Reviewer #1: Dear authors,

Content-wise, I am happy with the revised manuscript.

However, the manuscript needs to undergo another round of language editing. Revise the usage of past tense and past perfect tense in the manuscript. Grammatical errors need to be corrected:

1. Replace "gender" with "sex".

Response: All "gender" have been replaced with "sex"

2. Replace "scoping down" with "narrowing down".

Response: Amended as per advice

3. Line 86: There is no Reference no. 132.

Response: It is actually 13. There was a typo when the references were re-numbered.

4. Line 123

5. Line 136

6. Line 139

7. Line 156

8. Line 168

9. Line 253-254

10. Line 352

11. Line 355

12. Line 439

and throughout the manuscript.

Response: The authors have taken the assistance of grammar correcting software Grammarly and also taken the editing service from PM Proofreading Services. The certificate provided by them has been attached.

Reviewer #2: The author has addressed the issues raised by the current reviewer previously and the paper is now acceptable for publication.

Reviewer #3: All comments have been addressed. It is a pleasure to read the manuscript. It is a very interesting field to enhance the forensic investigation.

Our sincere gratitude to all the reviewers for your sincere contribution for the betterment of science. At the end, we hope that our sincere effort could address all the requirements of the editor and our revision would satisfy the imminent reviewers.

Sincerely yours,

M Tanveer Hossain Parash (corresponding author)
